# GSDME in cardiovascular diseases: research system and contemporary progress

**DOI:** 10.3389/fimmu.2025.1683116

**Published:** 2026-01-02

**Authors:** Zhenzhen Yang, Zilong Luo, Han Zhang, Junjie Zong, Pinyan Huang, Yuqing Niu, Cheng Zhou, Song Wang, Dan Zhang

**Affiliations:** 1 Department of Cardiovascular Surgery, Union Hospital, Tongji Medical College, Huazhong University of Science and Technology, Wuhan, Hubei, China; 2 Center for Translational Medicine, Union Hospital, Tongji Medical College, Huazhong University of Science and Technology, Wuhan, Hubei, China; 3 Key Laboratory of Organ Transplantation, Ministry of Education, NHC Key Laboratory of Organ Transplantation, Key Laboratory of Organ Transplantation, Chinese Academy of Medical Sciences, Wuhan, Hubei, China; 4 Cancer Center, Union Hospital, Tongji Medical College, Huazhong University of Science and Technology, Wuhan, Hubei, China; 5 Institute of Radiation Oncology, Union Hospital, Tongji Medical College, Huazhong University of Science and Technology, Wuhan, Hubei, China

**Keywords:** gasdermin E(GSDME), cardiovascular diseases, pyroptosis, gasdermin, cell death

## Abstract

Despite considerable advancements in treatment technologies in recent years, cardiovascular diseases still pose a significant threat to human health. Pyroptosis is a novel type of regulated cell death (RCD) associated with inflammation and innate immunity. Gasdermin E (GSDME), a key member of the gasdermin family, serves as a critical mediator of pyroptosis. Upon recognizing cellular stress or damage, GSDME can be activated through the classic caspase-3 cleavage pathway, releasing its N-terminal domain, which forms pores in the cell membrane to mediate pyroptosis and promote the release of inflammatory cytokines. Increasing evidence suggests that this process is closely associated with the progression of cardiovascular diseases, including atherosclerosis, myocardial infarction, nonischemic cardiomyopathy, and pulmonary arterial hypertension. These findings highlight the therapeutic potential of GSDME, with strategies targeting GSDME showing promising preclinical prospects. In this review, we introduce the structure and biological functions of GSDME, provide a brief overview of research strategies and experimental systems, and discuss recent scientific advances regarding GSDME in cardiovascular diseases. In addition, we explore the challenges currently facing the field and future research directions. A deeper understanding of the molecular mechanisms of GSDME in the cardiovascular system will provide a theoretical basis for the development of novel therapeutic strategies.

## 1 Introduction

Cardiovascular diseases (CVDs), including atherosclerosis (AS), myocardial infarction (MI), nonischemic cardiomyopathies, and hypertensive heart disease, remain the leading causes of death and disability worldwide (1). Despite significant advancements in diagnosis and treatment, the morbidity and mortality of CVDs remain persistently high, indicating that their pathological mechanisms have not yet been fully elucidated and highlighting the urgent need for the exploration of new therapeutic targets (2, 3). Cell death, as a core biological process maintaining tissue homeostasis, plays a crucial role in the pathogenesis and progression of CVDs when it is aberrantly activated or dysregulated (4). For a long time, apoptosis has been considered the predominant form of cardiovascular cell loss (5). However, accumulating evidence indicates that another highly inflammatory form of programmed cell death, pyroptosis, is significantly activated in various cardiovascular pathological processes and may have a more profound impact on disease progression (6).

Pyroptosis is an inflammatory form of programmed cell death mediated by the gasdermin (GSDM) family (7). The GSDM family is a group of proteins closely associated with cell death and is involved in various forms of programmed cell death, including apoptosis, pyroptosis and necrosis (8). In the human genome, the gasdermin protein family comprises GSDMA, GSDMB, GSDMC, GSDMD, GSDME and DFNB59, with GSDME attracting widespread attention because of its role in pyroptosis (9).

GSDME, also known as DFNA5, was initially discovered for its role in hereditary hearing loss (10), and in recent years, its identity as a key executor of pyroptosis has been extensively studied in the fields of tumors and infections (11). Like other GSDM family members, GSDME functions in cell death through its N-terminal gasdermin domain. Under conditions of cellular stress or damage, caspase-3 can activate GSDME, leading to the formation of pores at its N-terminus, disrupting cell membrane integrity, and resulting in the leakage of cellular contents and cell death (11). Unlike GSDMD, which is expressed primarily in immune cells, GSDME is expressed at basal levels in various parenchymal cells, including cardiomyocytes, vascular endothelial cells, and smooth muscle cells, where it can induce pyroptosis through membrane pore formation (8, 11). This widespread expression underscores its crucial role in mediating pyroptosis and its relevance in studying organ damage dominated by nonimmune cells, such as those in the heart and vasculature.

Given the important role of GSDME in different stages of specific CVDs, this review aims to systematically delineate the molecular characteristics and biological functions of GSDME, briefly provide an overview of research systems and experimental approaches related to GSDME, and focus on the latest research evidence of GSDME-mediated pyroptosis in major CVDs. We explore its specific cellular sources and pathological contributions and critically assess the limitations of the current research. Finally, we summarize the exploratory outcomes of pharmacological interventions targeting GSDME in CVD models to target the GSDME-induced pyroptosis pathway in preventing and treating CVD.

## 2 Molecular characteristics and biological functions of GSDME

### 2.1 Structural features and expression

The wild-type GSDME gene consists of 10 exons, which encode a protein of 496 amino acids with a molecular weight of 55 kDa, encoded by a gene located on chromosome 7p15 (12). As a key member of the GSDM family, GSDME has highly conserved structural features: an N-terminal pore-forming domain (approximately exons 2–6), a hinge region (exons 6–7) and a C-terminal autoinhibitory domain (exons 7–10). GSDME forms a stable autoinhibited conformation through intramolecular electrostatic interactions (13). In its resting state, this intramolecular interaction prevents membrane localization and oligomerization of the NTD, maintaining the protein in an inactive state (11). Notably, when cells are subjected to specific stimuli, caspase-3 or granzyme B cleave the protein at the aspartate residue (Asp270) located in the linker region, thereby separating the two domains and inducing pyroptosis (14). In line with this, familial DFNA5 hearing loss mutations that remove the C-terminal portion of GSDME lead to a constitutively active N-terminal fragment, resulting in spontaneous cochlear cell death (14). In other words, the mutant GSDME behaves as if it were in a permanent “cleaved” form, highlighting the critical role of the C-terminal domain in preventing unnecessary cytotoxicity (10, 15). This structural arrangement is similar to that of GSDMD and other gasdermins, emphasizing the conserved mechanisms of self-inhibition and activation across the entire family (16).

GSDME is expressed in a wide range of human tissues, albeit with some variability (17). Notably, under normal physiological conditions, GSDME is highly expressed in organs such as the placenta, small intestine, and lungs, whereas its expression is relatively low in the heart, skeletal muscles, and cerebral cortex (14). However, under pathological conditions, the expression of GSDME can change dramatically. In the context of cardiovascular diseases, studies have shown that the expression of GSDME in macrophages within atherosclerotic plaques is significantly greater than that in normal vascular tissue and that the levels of activated N-GSDME fragments are notably elevated (18). Single-cell RNA sequencing confirmed that in advanced human carotid plaques, the colocalization coefficient of GSDME with macrophage markers (CD68, CD163, and CD16) exceeds 90%, suggesting its potential role in macrophage-mediated plaque destabilization (18).

### 2.2 Activation and regulatory mechanisms

As mentioned above, GSDME is a latent pore-former that requires proteolytic activation. The key activating protease is caspase-3, the executioner caspase of apoptosis (11). Importantly, caspase-3 is normally associated with apoptosis, but when GSDME is abundant, caspase-3 activation switches the mode of death from apoptosis to pyroptosis (19, 20). Experimental evidence has shown that cells with high GSDME expression do not neatly fragment into apoptotic bodies upon caspase-3 activation; instead, they swell and rupture, manifesting a pyroptotic morphology (14). Conversely, if GSDME expression is low or absent, caspase-3 activation occurs via the classical apoptotic route, with cell shrinkage and membrane integrity maintained until phagocytic clearance (14). Jiang et al. reported that caspase-3 is a common executor in both pathways and that the expression level of GSDME determines the outcome (21). This mechanism has been observed in various cellular contexts, where external stressors or cytotoxic stimuli trigger pyroptosis via caspase-3 cleavage of GSDME (22). In addition to caspase-3, studies have indicated that inflammatory proteases from immune cells can also activate GSDME. Granzyme B (GzmB), a serine protease released by cytotoxic T lymphocytes and NK cells, was found to directly cleave GSDME after Asp-270, the same site used by caspase-3 (20). GzmB not only cleaves GSDME but also proteolytically activates caspase-3 itself, creating a two-pronged attack on target cells (23). As a result, when killer lymphocytes attack a GSDME-expressing cell, pyroptosis can be triggered: Gzm B enters the target cell, cleaves GSDME to unleash the pore-forming domain, and simultaneously amplifies the caspase-3 activation cascade to ensure robust GSDME cleavage (24). This mechanism has been shown to contribute to the immunological killing of tumor cells; if the cancer cell expresses GSDME, an NK or CD8+ T-cell can induce it to undergo explosive pyroptotic death, thereby alerting the immune system and possibly improving antigen release for cross-priming (25, 26). However, its relevance in cardiovascular cells remains speculative and needs further investigation. Interestingly, recent studies have demonstrated that GSDME can be activated through nonproteolytic cleavage-dependent pathways. For example, UV-C irradiation activates cleavage-independent pyroptosis via full-length GSDME through synergistic PARP1/PARP5-mediated PARylation and cytochrome c-driven cardiolipin peroxidation (27).

Given the core position of GSDME in pyroptosis, its expression and activity are tightly regulated at multiple levels, including the epigenetic, transcriptional and posttranslational control. One major mode of regulation is epigenetic silencing. The GSDME gene promoter contains CpG islands that are prone to DNA methylation, especially in cancer cells (28). Hypermethylation of the GSDME promoter prevents transcription factor binding and effectively silences the gene (29). Studies in the past decade have revealed frequent GSDME promoter hypermethylation in a variety of cancers, including breast, gastric, and colorectal tumors (30). Although initially characterized in cancer, such methylation-based repression may also influence GSDME expression in nonmalignant tissues exposed to chronic metabolic or inflammatory stress. Clinically, GSDME methylation is being explored as a potential biomarker. For example, one study revealed that a specific CpG site in the GSDME promoter was highly methylated in breast tumors compared with normal tissue, suggesting its diagnostic application (31). At the transcriptional level, GSDME expression is controlled by several factors. For instance, p53 directly binds to the GSDME promoter and induces its expression following DNA damage, linking genotoxic stress to cell death responses (32). Other transcription factors have also been implicated. For example, STAT3, a transcription factor activated by inflammatory cytokines, was reported to increase GSDME expression in vascular endothelial cells during atherosclerosis (18). Chronic inflammation in atherosclerotic lesions leads to increased GSDME levels, presumably via the binding of STAT3 to the GSDME promoter (18). Another regulator is the zinc-finger factor specificity protein 1 (Sp1). A recent study identified Sp1 as a positive transcriptional regulator of GSDME: Sp1 can bind a GC-rich motif in the GSDME core promoter and drive robust promoter activity (33). Overexpressing Sp1 upregulated GSDME, whereas knocking down Sp1 or using an Sp1 inhibitor reduced GSDME mRNA levels (33). Interestingly, this study also suggested that the regulatory process of SP1 has synergistic effects on STAT3 and antagonistic effects on DNA methylation (33). This finding points to a complex regulatory module: inflammatory signaling and constitutive factors try to turn GSDME on, but epigenetic methylation may lock it off in certain cells. At the posttranslational level, GSDME activity can be modulated by protein modifications. A striking example is the role of phosphorylation. Rogers et al. reported that the GSDME N-terminal domain has a conserved threonine near its N-terminus that can be phosphorylated, whereas a phosphomimetic mutation at this site inhibited the pore-forming activity of GSDME-N (34). Supporting this, Ai et al. found that through GlcNAc-6P -mediated activation of AMPK phosphorylates GSDME at Thr6, preventing its cleavage and pyroptosis (35). Functionally, this regulation protected tissues from chemotherapy-induced tissue injury both in mouse models and patients (35). Additionally, alternative cleavage sites or proteases might modulate outcomes. For example, inflammatory caspases generally do not cleave human GSDME, but under some conditions, caspase-8 can cleave gasdermin (36). Overall, the cell’s decision to undergo GSDME-mediated pyroptosis is governed by a balance of these regulatory inputs: gene methylation and transcriptional activation determine how much GSDME protein is present, and protease activation and posttranslational modifications determine whether the GSDME that is present is activated to form membrane pores.

## 3 Research systems and experimental approaches of GSDME

### 3.1 Molecular and cellular models and experimental approaches

To investigate the molecular mechanisms of GSDME activation and function, researchers employ various molecular and cellular models, including cardiomyocytes, vascular endothelial cells, as well as vascular smooth cells (18, 37, 38). GSDME expression or activation can be experimentally modulated in these models, which provide controllable settings to dissect the upstream signals, cleavage events, and downstream effects of GSDME-mediated pyroptosis.

Within these models, a broad toolkit of molecular assays is applied to characterize GSDME expression, localization, and function. Protein expression and cleavage are typically examined via Western blotting. For example, cell or tissue lysates are probed for full-length GSDME (~55 kDa) and its cleaved N-terminal fragment (~30 kDa) via specific antibodies (11). An increase in the GSDME-N band, often alongside active caspase-3, is a hallmark of GSDME activation (39). As mentioned above, western blotting verified drug-induced GSDME cleavage in cancer cells and patient samples (11). Similarly, the mRNA levels of GSDME were measured via quantitative PCR (qPCR) to assess transcriptional regulation (40). In atherosclerotic mouse aortas, qPCR revealed higher Gsdme mRNA in diseased than in normal diet conditions (18). These RNA assays complement protein data to confirm changes at the transcriptional level.

Immunodetection methods pinpoint the cellular and subcellular localization of GSDME. Immunofluorescence (IF) and immunohistochemistry (IHC) with anti-GSDME antibodies were used to visualize where GSDME is expressed and activated. In cultured cells, IF can reveal the redistribution of GSDME upon cleavage; for example, confocal microscopy of cells expressing GSDME-N–EGFP revealed its translocation from the cytosol to punctate membranes (34). Colocalization with organelle markers is used to identify targets of the GSDME-N fragment. Moreover, the study revealed that GSDME-N puncta overlapped with MitoTracker-stained mitochondria, and overlapping regions presented a diminished MitoTracker signal (34). These findings provide visual evidence that cleaved GSDME-N targets and permeabilizes mitochondria. In tissues, IHC is critical for mapping GSDME expression (41). As mentioned earlier, Wei et al. stained human carotid endarterectomy specimens and reported strong GSDME immunoreactivity in lesional cells versus minimal staining in normal arteries. They further used *in situ* proximity ligation assays (PLAs) to detect GSDME–caspase-3 complexes in plaques, confirming that caspase-3 cleavage of GSDME occurs *in vivo* (18).

Loss- and gain-of-function genetic approaches are cornerstone tools for interrogating GSDME. RNA interference (siRNA/shRNA) can transiently knock down GSDME to test its necessity in a given phenotype. For example, silencing GSDME in cancer cell lines has been shown to increase their clonogenic survival and invasiveness, which is consistent with the loss of pyroptotic cell death constraints (42). Conversely, exogenous expression of GSDME in GSDME-low tumor cells suppressed their growth and viability (43). Permanent gene editing via CRISPR/Cas9 has allowed the generation of GSDME knockout cell models. Researchers often compare wild-type and GSDME-knockout clones side-by-side; for example, in Hepa1–6 cells, CRISPR deletion of GSDME abolished miltirone-induced LDH release and plasma membrane ballooning, indicating that GSDME amplifies the cell death signal (44). CRISPR has also been used to reintroduce GSDME (or mutants) into knockout backgrounds to rescue function and test mutant phenotypes (20). Additionally, overexpression systems (plasmid or viral transduction) are employed to drive GSDME or its fragments in the cells of interest (45). Transient overexpression of GSDME-N is sufficient to induce pyroptotic death in 293T cells and other cells, which is useful for studying pore-forming activity in isolation (34).

In summary, a combination of biochemical assays, imaging techniques, and genetic perturbations is used *in vitro* to elucidate how GSDME is activated and how it executes pyroptosis at the cellular level. These approaches firmly establish that GSDME cleavage by caspase-3 (or granzyme B) liberates a cytotoxic N-terminal domain that oligomerizes in membranes to drive lytic cell death.

### 3.2 Animal models and *in vivo* experimental approaches

To dissect GSDME’s role in cardiovascular pathology, researchers use genetically modified animals and disease models. Animal models, particularly genetically engineered mice, are indispensable for investigating the functions of GSDME in physiology and disease. GSDME-knockout mice (Gsdme^−/−^) are a primary tool used to define the *in vivo* role of this protein. These mice are viable and develop normally, as GSDME is largely dispensable in the absence of pathological triggers (46). However, under stress conditions, Gsdme^−/−^ mice exhibit remarkable resistance to injuries, which are exacerbated by pyroptosis in wild-type mice (47). For example, Wan et al. reported that Gsdme^−/−^ mice were protected from H7N9 virus infection-induced alveolar epithelial cell damage and pulmonary cytokine storm (48). Similarly, in the context of cancer immunotherapy, GSDME deficiency has been shown to be beneficial: in a model of checkpoint inhibitor-induced myocarditis, mice lacking GSDME were largely protected from the fulminant immune-mediated cardiac inflammation that afflicted wild-type mice (49). In another striking example, a recent study of silicosis demonstrated that, compared with single knockouts, double-knockout mice (Gsdme^−/−^/Gsdmd^−/−^) presented markedly attenuated lung pathology, revealing that caspase-3/GSDME-dependent pyroptosis can compensate when the canonical inflammasome pathway is blocked (50). Together, these models underscore that GSDME is a key “executioner” whose presence can worsen tissue injury by converting what would be apoptosis into inflammatory necrosis.

Transgenic and knock-in models have also been employed to investigate GSDME, especially in the context of inherited disease. GSDME was first identified as the gene mutated in DFNA5 autosomal-dominant deafness (10, 51). Deafness-causing mutations produce a truncated GSDME that is thought to be a constitutively active N-terminal fragment (12). To mimic this, Xiao et al. introduced a human DFNA5 mutation into mice and observed progressive hearing loss with degeneration of cochlear hair cells and spiral ganglion neurons (52). Importantly, hair cell death in these mice is characterized by pyroptosis, establishing a mechanistic link between GSDME-mediated pyroptosis and hearing loss. In addition to its role in hearing loss, studies using GSDME-targeted delivery in mouse models have also demonstrated its significant involvement in susceptibility to inflammatory bowel disease, ulcerative colitis, and other genetically predisposed autoimmune disorders (53–55). Furthermore, GSDME knock-in models have proven instrumental in elucidating the pivotal role of GSDME-mediated pyroptosis in the physiological and pathological processes of various common diseases. For example, Mao et al. revealed that the overexpression or knockout of GSDME plays a crucial role in DON-induced pyroptosis and inflammation in murine hepatic cells (56). Similarly, mouse models harboring specific GSDME mutations have been developed to mimic the pyroptotic process, enabling the investigation of the potential role of GSDME in neurodegenerative diseases such as Alzheimer’s disease (57). We discuss cardiovascular diseases in detail in the next section. These models are equally indispensable in the field of cancer (58, 59).

Taken together, transgenic, knockout, and precise gene knock-in models collectively form the core research tools for understanding the biological functions of GSDME and its pathogenic and therapeutic mechanisms in major diseases such as genetic disorders, cancer, and inflammation, thereby providing a solid theoretical foundation for the development of targeted therapeutic strategies targeting the GSDME pathway.

### 3.3 Clinical samples and omics-based analyses

To bridge bench findings with human disease relevance, analyses of clinical tissues, circulating biomarkers, and publicly available transcriptomic and methylomic datasets enable evaluation of GSDME expression, regulation, and disease association. Integration of transcriptomic and methylomic data provides complementary insights into how GSDME expression correlates with cancer-related and apoptotic gene networks (60) (61). Moreover, profiling of circulating cell-free DNA allows assessment of GSDME methylation and expression in patients, offering potential biomarkers that connect molecular activation with early cancer diagnosis and treatment efficacy prediction (62).

Clinical sample analysis has also shed light on GSDME in nonmalignant diseases. Emerging evidence from patient-derived biospecimens revealed that dysregulated GSDME activation contributes to the pathogenesis of multiple inflammatory and degenerative conditions. In sepsis, elevated plasma levels of GSDME-N fragments correlate with disease severity and cytokine storms (63). Furthermore, in a human proximal renal tubular epithelial cell line, researchers demonstrated that chemotherapy drug-induced nephrotoxicity is mediated through the ROS-JNK-caspase 3-GSDME signaling pathway. Gene silencing of GSDME significantly alleviated cisplatin- or doxorubicin-induced pyroptosis in renal tubular epithelial cells (64). Through bioinformatics analysis, researchers identified Caspase-8 as a key gene involved in Alzheimer’s disease pathology and pyroptosis (65). It regulates the expression of GSDME, influencing the level of pyroptosis and linking it to the pathological progression of tau protein expression (65). In cardiovascular tissues, investigators have begun examining human biopsies for GSDME activation markers as well. Compared with that in normal arterial tissue, GSDME protein expression is markedly elevated in advanced atherosclerotic plaques from human carotid arteries (18). Moreover, in patients with pulmonary arterial hypertension (PAH), lung tissue analyses revealed that GSDME levels were higher in the vascular endothelial cells of PAH patients than in those of healthy controls, and this finding was correlated with endothelial apoptosis/pyroptosis in this disease (66). Mechanistic work has shown that a proteolytic factor elevated in PAH (cathepsin L) can trigger caspase-3–GSDME activation, leading to endothelial cell pyroptosis and loss, thus contributing to vascular remodeling (66). These findings from patient tissues align with the animal and cell culture data, reinforcing the clinical relevance of GSDME-driven cell death.

Overall, clinical sample analysis has confirmed the clinical importance of GSDME in both cancer and nonmalignant diseases, particularly in cardiovascular diseases, highlighting its potential as a biomarker and therapeutic target in various pathological conditions. Simultaneously, with the rise of big data, future research will likely integrate genomics, epigenomics, transcriptomics, and even single-cell proteomics from patient samples to build a comprehensive picture of where GSDME is expressed and active in human health and disease.

## 4 Contemporary research progress of GSDME in cardiovascular diseases

In this section, we examine evidence for the involvement of GSDME in major cardiovascular pathologies. We focus on diseases with high prevalence and mortality, notably atherosclerosis (and its complications), myocardial infarction, nonischemic cardiomyopathies (including dilated cardiomyopathy, hypertrophic cardiomyopathy, diabetic cardiomyopathy and toxic cardiomyopathy) and hypertension-induced cardiac damage. For each, we summarize key findings from recent studies and indicate the consensus or controversies regarding the role of GSDME.

### 4.1 Atherosclerosis

Atherosclerosis is a chronic inflammatory disease of large- and medium-sized arteries characterized by lipid-rich plaques in the arterial wall (67). Plaque rupture can precipitate acute cardiovascular events (MIs, stroke), making atherosclerosis a leading cause of CVD mortality (68). Inflammation and macrophage death within plaques drive plaque growth and instability, particularly the formation of a necrotic core of dead cells and debris (69). Pyroptosis has been increasingly recognized as a form of macrophage death contributing to these processes (18). As mentioned earlier, GSDME is detected in multiple cell types in plaques, including endothelial cells (ECs), macrophages and vascular smooth muscle cells (VSMCs), which are key players in atherogenesis (18).

ECs line the arteries, and their dysfunction is a trigger for plaque initiation (70). Xie et al. confirmed that endothelial cells in atheroprone areas express GSDME and that their activation is correlated with vascular inflammation (38). Specifically, in cultured human endothelial cells, oxidized LDL can activate caspase-3 and GSDME, inducing EC pyroptosis and the release of inflammatory mediators (18). A novel mechanism has been proposed: GSDME-N-terminal fragments can damage EC mitochondria, leading to the release of mitochondrial DNA (mtDNA) into the cytosol (38). Cytosolic mtDNA activates the cGAS-STING pathway, a DNA-sensing immune pathway, which in turn drives the expression of type I interferons and NF-κB-dependent inflammatory genes (71). Moreover, in the context of GSDME deficiency, partial restoration of the phosphorylation and activation of the STING pathway was noted upon its activation (38). Thus, GSDME links endothelial injury to innate immune activation in atherosclerosis through mtDNA-STING signaling.

Macrophages play a crucial role in the progression of atherosclerosis by engulfing oxidized low-density lipoprotein to form foam cells, thereby maintaining cholesterol homeostasis (72). Interestingly, researchers have reported that GSDME expression is significantly elevated in macrophages within atherosclerotic lesions and contributes to the progression of atherosclerosis through pyroptosis, which can be significantly inhibited by the ablation of GSDME (18). Furthermore, silencing endogenous STAT3 reduced GSDME expression, further suggesting a potential therapeutic approach for atherosclerosis (18). A recent study demonstrated for the first time that GA2, a subclass of gangliosides that significantly accumulate in the arteries and plasma of atherosclerotic patients, regulates the activation of the Casp4/11 signaling pathway, which controls the tBID-mediated cytochrome C-Casp9-Casp3-GSDME pathway, ultimately accelerating intimal hyperplasia following arterial injury (73).

VSMCs, as key participants in both early-stage atherosclerosis and late-stage atherosclerosis, are able to populate both the fibrous cap and plaque core and have different effects on atherogenesis through the adoption of different cellular phenotypes (74). Researchers have confirmed the presence of GSDME in VSMCs (75). While GSDME was implicated in apoptosis in this study, it can be inferred that pyroptosis may also play a role in this process. As atherosclerosis progresses, vascular smooth muscle cells undergo phenotypic transformation, which promotes artery calcification (AC) and increases the risk of cardiovascular events (76). Recently, Yang et al. reported that the Caspase-3/GSDME axis promotes the development of AC (77). Previous studies reported that HMGB1 is regulated by GSDME (78). Researchers reported that in a constructed AC mouse model, HMGB1 levels were significantly elevated, and the Caspase-3/GSDME axis was markedly activated (77). Conversely, specific knockdown of GSDME in VSMCs significantly inhibited inflammation and effectively alleviated the progression of calcification in mice (77).

In summary, an increasing body of research suggests that GSDME influences the progression of atherosclerosis by promoting pyroptosis in key cells. These findings highlight the potential of targeting GSDME as a promising strategy for stabilizing atherosclerotic plaques.

### 4.2 Myocardial infarction

Myocardial infarction is typically caused by acute thrombotic occlusion of a coronary artery (79). The resulting ischemia and subsequent reperfusion induce the death of cardiomyocytes in the affected area (80, 81). MI initiates an intense inflammatory response in the heart and can lead to adverse ventricular remodeling or heart failure over time (82, 83). Cardiomyocyte death in MI is thought to occur via ischemic necrosis and apoptosis (84). However, recent evidence suggests that pyroptosis contributes significantly to myocardial injury during MI and ischemia–reperfusion (I/R) (85). Notably, caspase-1- and GSDMD-mediated pyroptosis in cardiomyocytes and infiltrating immune cells has been shown to increase infarct size and worsen cardiac dysfunction (86). The role of GSDME in MI is a burgeoning area of research, intersecting with both the acute phase of cardiomyocyte death and the subacute phase of cardiac remodeling.

During acute MI, rapid inflammatory and cell death responses occur (87). Studies using animal I/R models and cardiomyocyte-specific GSDMD knockout models have demonstrated that GSDMD-driven pyroptosis in cardiomyocytes is a key event in acute I/R injury and that its inhibition can reduce infarct size (88, 89). Interestingly, GSDME levels in the heart did not significantly change in the very early phase after I/R. These findings suggest that during the first hours of reperfusion, the inflammatory caspase-1/11 pathway may be more predominant, whereas the caspase-3/GSDME pathway is less active. This finding is consistent with the conclusion proposed by Guo et al., which suggests that GSDME has high accuracy in predicting the risk of acute myocardial infarction (AMI) and offers new potential pathways for targeted therapy of AMI (90). Liu et al. reported that pyroptosis-related genes were upregulated approximately 24 hours post-MI, whereas the initial 6 hours post-MI were marked by immune activation and likely apoptotic signaling (91). Consistent with these findings, early intervention with a caspase-1 inhibitor (VX-765) within hours of MI reduced IL-1β release and infarct size and preserved cardiac function (91). Thus, immediately after MI, GSDMD-mediated pyroptosis appears to be the primary contributor to cell death and inflammation, whereas GSDME might not yet be fully engaged.

As the infarct evolves, conditions for GSDME activation may arise. Apoptosis is known to occur in the border zone and remote myocardium in days following MI due to oxidative stress, cytokines, and strain on surviving cardiomyocytes (92). If GSDME is present in these cells, caspase-3 activation during apoptosis can initiate pyroptosis. In a murine MI model, Zheng et al. reported that doxorubicin-triggered cardiac injury led to caspase-3 cleavage of GSDME and pyroptotic death of cardiomyocytes (37). By analogy, in MI, apoptotic cardiomyocytes could undergo GSDME-mediated pyroptosis, thereby converting what would be a noninflammatory cell clearance into an inflammatory event that recruits more leukocytes into the myocardium. Indeed, evidence of GSDME-N-terminal fragment formation has been detected in myocardial tissue after infarction in some studies. A recent experimental therapy study provided indirect support: kaempferol (a natural flavonoid) was found to protect cardiomyocytes from hypoxia/reoxygenation (an *in vitro* MI model) by promoting O-GlcNAcylation of GSDME, which inhibited its pyroptotic activity (93). Kaempferol treatment in an acute MI mouse model reduced myocardial injury and notably decreased cleaved GSDME-N levels in the heart (93). This finding implies that in the days following MI, GSDME is active in cardiomyocytes and contributes to ongoing cell death and that strategies to inhibit GSDME can mitigate damage.

MI triggers an influx of neutrophils and monocytes into the heart, which removes dead cells but can also cause collateral damage (87). These immune cells themselves can undergo pyroptosis, which releases more IL-1β and propagates inflammation (86, 94). While GSDMD is mainly responsible for neutrophil pyroptosis, GSDME may also play a role in monocyte/macrophage death post-MI. Clinical evidence has shown that elevated GSDMD expression in the peripheral blood mononuclear cells (PBMCs) of acute MI patients is correlated with systemic inflammation (95). GSDME expression in PBMCs has not been reported widely, but caspase-3 activity is often increased systemically after MI due to circulating stress signals (96). It is conceivable that a subset of monocytes or lymphocytes with high GSDME could undergo pyroptosis in the post-MI inflammatory milieu, although direct evidence is still emerging.

Taken together, during acute MI, GSDME initially plays a lesser role. However, as apoptosis occurs, GSDME likely becomes an important amplifier of cell death and inflammation. By converting late-stage apoptosis to pyroptosis, GSDME can exacerbate myocardial injury and impede optimal healing. This finding suggests a time-dependent approach: targeting inflammasome/GSDMD early and GSDME-mediated pathways slightly later might reduce total cell loss.

### 4.3 Non-ischemic cardiomyopathies

Non-Ischemic cardiomyopathies are diseases of the heart muscle that are not due to coronary artery disease or hypertension (97). These conditions include dilated cardiomyopathy (DCM), hypertrophic cardiomyopathy (HCM), and specific types, such as diabetic cardiomyopathy and toxic cardiomyopathy (97). Many ultimately lead to heart failure or arrhythmias (98). They often involve genetic mutations, metabolic stress, or toxic insults that cause cardiomyocyte dysfunction and death (99, 100). Inflammation is variable; for example, HCM is largely genetic and not primarily inflammatory, whereas myocarditis is by definition immune-mediated (97). Here, we focus on intrinsic cardiomyopathies and highlight myocarditis/arrhythmia, which overlap with cardiomyopathy in some contexts.

DCM is characterized by enlargement of the ventricles and systolic dysfunction (101). It can result from genetic mutations, viral myocarditis, alcohol abuse, etc. (102) In DCM, there is evidence of ongoing low-level myocarditis; immune cells can be found in the myocardium, and elevated cytokines are common (103). Pyroptosis might be active in DCM; for example, one study of failing DCM hearts reported the upregulation of caspase-1 and IL-18 (104). However, direct links to GSDME are scarce. It is reasonable to hypothesize that if apoptotic signals are present in DCM, GSDME could mediate some cardiomyocyte death. A bioinformatic analysis of HCM by Tang et al. revealed that pyroptosis-related genes, such as GSDMD, are part of the gene network dysregulated in HCM (105). In HCM, the dominant pathology is hypertrophy and fibrosis; cell death is not a major early feature, but in late-stage HCM, small vessel disease can cause microinfarcts and myocyte apoptosis (106). The role of GSDME in HCM has not been studied, but given that caspase-3 has been found to be activated in areas of fibrosis in HCM hearts, one might suspect that GSDME contributes to myocyte loss and inflammation in advanced cases. This remains speculative until more data emerge.

Diabetic cardiomyopathy refers to heart muscle dysfunction caused by diabetes, which is independent of coronary disease. High glucose and lipid toxicity lead to oxidative stress in cardiomyocytes and the activation of NF-κB and the NLRP3 inflammasome (107). Multiple studies in diabetic rodents have shown increased myocardial caspase-1, IL-1β, and GSDMD cleavage, indicating that pyroptosis occurs in the diabetic heart (108). In parallel, there is often caspase-3 activation via hyperglycemia-induced apoptosis pathways (109). If GSDME is present, apoptotic cardiomyocytes may undergo pyroptosis. A recent Frontiers in Physiology review noted that while GSDMD’s role in DCM has been established, “no study has proven that caspase-3 induces GSDME in cardiomyocytes treated with high glucose” (110). This indicates a gap in knowledge. This review posed the question of whether ROS might be the link that increases caspase-3 and thus GSDME cleavage. Given the parallels to other systems, it is plausible that in diabetic hearts, excessive ROS trigger the release of cytochrome c from mitochondria, the activation of caspase-3, and then GSDME-mediated pyroptosis (38). Notably, Yuan et al. identified MGO as a critical factor in the development of diabetic vascular complications and demonstrated that it can induce pyroptosis in HUVECs (human umbilical vein endothelial cells) through the ROS/Caspase-3/GSDME signaling pathway (111). These findings suggest that GSDME inhibition could be cardioprotective in diabetes. Research to test this hypothesis could involve the use of GSDME knockout mice in a diabetes model to determine whether heart function is preserved. To date, that experiment has not been reported.

For toxic cardiomyopathy, we previously discussed doxorubicin-induced pyroptosis. To reiterate, DOX-induced cardiotoxicity is directly linked to GSDME via caspase-3. GSDME knockout was shown to reduce cardiomyocyte death in DOX models (37). Another recent study on DOX-induced cardiotoxicity revealed that GSDMD can also be activated, causing pyroptosis, and that inhibiting GSDMD alleviated DOX-induced heart failure (112). These findings suggest that DOX might activate multiple gasdermins: GSDME in cardiomyocytes and GSDMD in infiltrating immune cells or cardiac macrophages. Thus, combined blockade of GSDMD and GSDME might protect against chemopathic effects. Additionally, studies have investigated the role of GSDMD in sepsis-induced myocardial dysfunction, revealing that the accumulation of GSDMD-NT in cardiac tissue leads to mitochondrial dysfunction and excessive oxidative stress, which subsequently activates the NLRP3 inflammasome (83). Although there is currently no direct evidence linking GSDME to sepsis-induced cardiomyopathy, existing studies have shown that GSDME plays a role in sepsis-associated damage to other organs (113, 114), which may suggest the potential involvement of GSDME in sepsis-induced myocardial injury. Future research should further explore the specific mechanisms underlying the role of GSDME in sepsis-induced cardiomyopathy to assess its potential as a therapeutic target.

In summary, nonischemic cardiomyopathies present scenarios of chronic cardiomyocyte stress and death where GSDME could contribute, especially when apoptotic pathways are triggered. While GSDMD-driven pyroptosis has clearer evidence in many of these conditions, the involvement of GSDME is an emerging concept with unlimited potential.

### 4.4 Pulmonary arterial hypertension

Pulmonary arterial hypertension is a severe cardiovascular disease caused by various etiologies and is characterized by increased pulmonary artery pressure, which leads to an increased right ventricular load and may eventually result in right heart failure (115). The progression of PAH is driven by a series of complex pathological processes, with vascular endothelial injury being a key event (116). Increasing evidence from recent studies highlights the critical role of inflammation and cell death in endothelial injury (117). Genetic ablation or pharmacological inhibition of the NLRP3 inflammasome significantly attenuated the endothelial cell injury induced by ROS and other danger-associated signals, thereby mitigating inflammation and vascular dysfunction (118). Notably, the activation of GSDME induces programmed cell death in various inflammatory diseases, including PAH, which is characterized by profound vascular pathological changes (66). In a rat model, silencing the expression of the GSDME gene significantly reduced ventricular systolic pressure and alleviated the degree of right ventricular hypertrophy (119). In the same study, researchers reported that knockout of the NLRP3 gene significantly downregulated the expression levels of pyroptosis-related proteins (119). These findings suggest that inflammation-induced GSDME-mediated pyroptosis may be one of the underlying mechanisms of endothelial injury in PAH, suggesting novel insights into the inflammatory mechanisms underlying PAH therapy. In fact, Wu et al. established the critical role of caspase-4/11 in regulating pulmonary vascular dysfunction through the pyroptosis pathway, which accelerated the progression of PAH (120). Interestingly, their findings also revealed that caspase-4-induced pyroptosis involves the caspase-3–GSDME axis, which aligns with our hypothesis. Furthermore, recent studies have revealed for the first time that the lysosomal protease cathepsin L promotes the development of PAH by degrading BMPR2, thereby inducing endothelial cell injury through the BMPR2/caspase-3/GSDME-mediated pyroptosis pathway (66). This finding not only further strengthens the association between GSDME and PAH but also offers novel insights into the diagnosis and therapeutic strategies for PAH.

During the progression of PAH, the abnormal proliferation and migration of VSMCs are key mechanisms driving vascular remodeling (121). As previously mentioned, GSDME may be involved in secondary necrosis and the release of proinflammatory cytokines following VSMC apoptosis (75). Although the precise contribution of GSDME to pulmonary vascular remodeling remains to be elucidated, given its potential role in chronic inflammatory environments, it can be hypothesized that GSDME-mediated cell death may exacerbate endothelial injury, thereby promoting the migration and fibrotic response of vascular smooth muscle cells.

**Figure 1:**
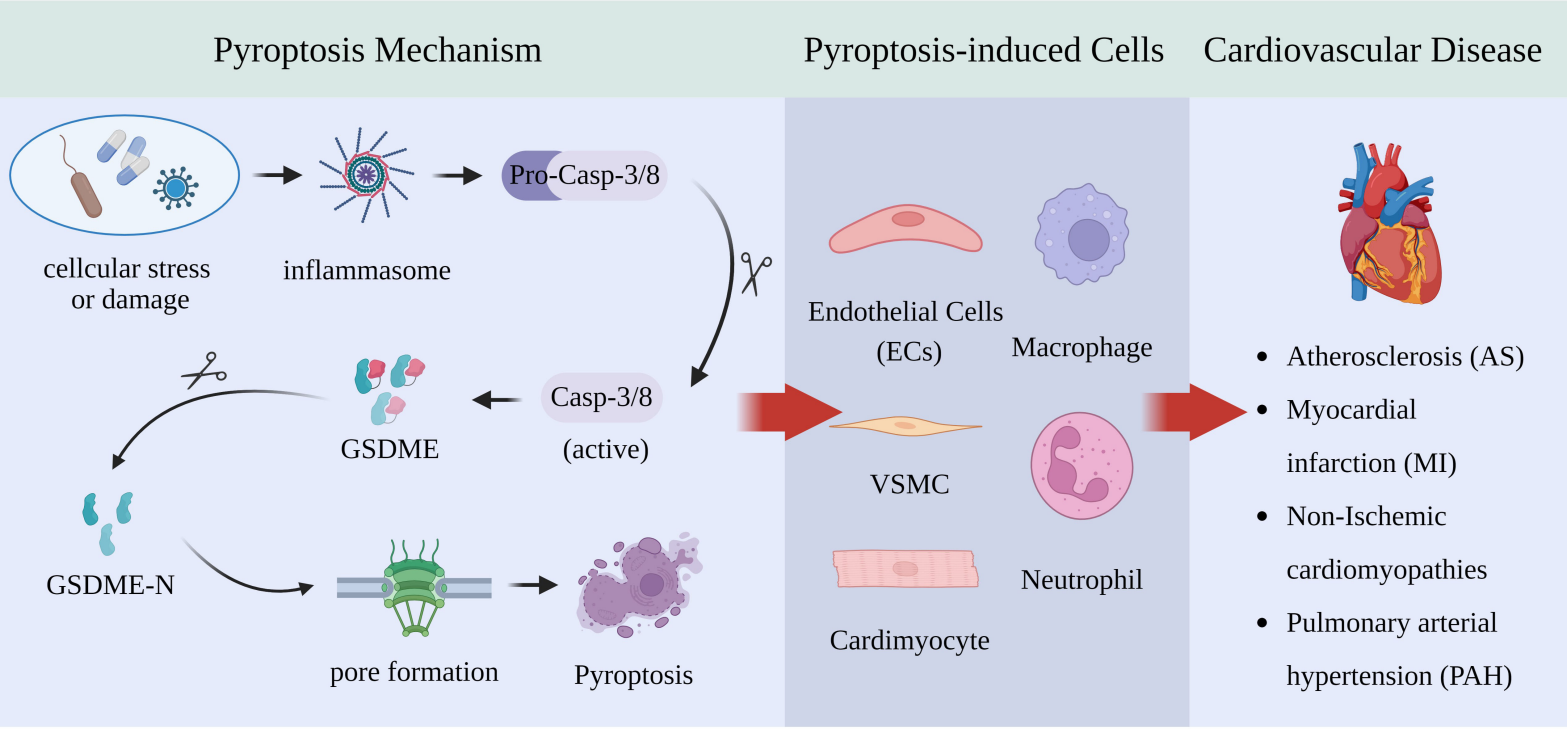
GSDME-expressing cells and pyroptotic mechanisms in cardiovascular diseases.

**Figure 2:**
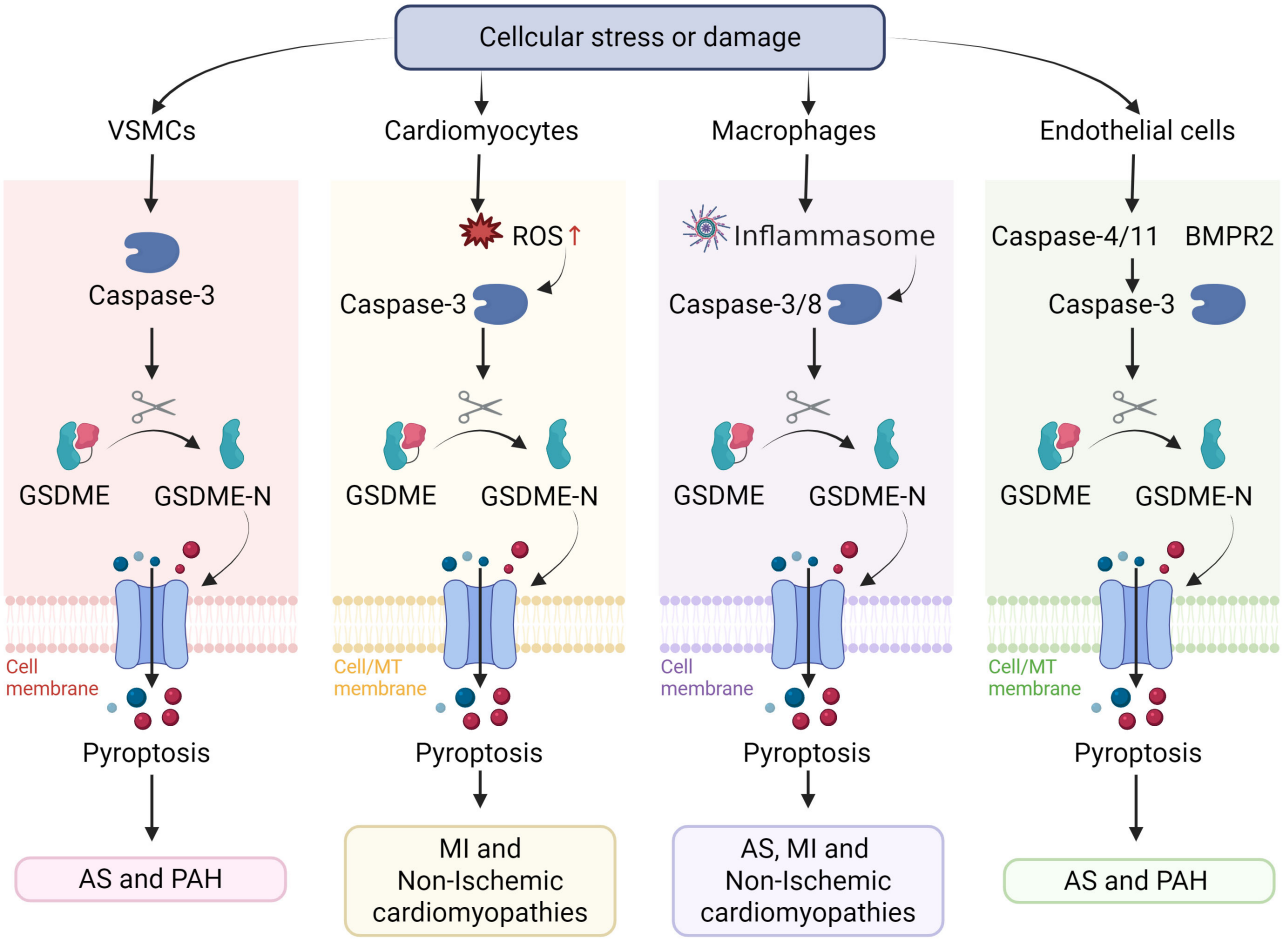
GSDME-induced pyroptosis in different cell types. MT membrane, mitochondrial membrane.

In summary, pyroptosis is harmful to PAH. The specific role of GSDME is still speculative but plausible. Because hypertensive damage occurs over a long period, one could imagine that even a modest contribution of GSDME-mediated pyroptosis could cumulatively worsen outcomes. Fortunately, existing studies have revealed that modulating the activation of GSDME can effectively alleviate the pathological progression of PAH. Future studies are needed to explore the specific mechanisms of GSDME in PAH and to develop corresponding pharmacological interventions, with the aim of providing new directions for clinical treatment.The cardiovascular diseases and their associated cellular pyroptosis mechanisms mentioned in the text are shown in **Figures 1** and **2**.

## 5 Limitations and perspectives

GSDME has emerged from relative obscurity (known first as a deafness gene) to become recognized as a significant mediator of inflammatory cell death in cardiovascular disease (122). This review highlights that GSDME-mediated pyroptosis contributes to the pathogenesis of atherosclerosis, myocardial infarction, nonischemic cardiomyopathies and hypertension-induced cardiac damage.

Despite these advances, several aspects of GSDME in CVD remain incompletely understood (1): Precise crosstalk between GSDME and GSDMD in the heart: under what conditions does one dominate over the other, and do they compensate if one is absent? This has implications if we target only one. Notably, apoptosis-related signaling and GSDME in the heart are “less understood” than inflammasome pathways are (2). Cell type-specific roles of GSDME: Most studies have focused on macrophages and endothelial cells, but what about fibroblasts or smooth muscle cells in vessels? More work is needed to delineate the function of GSDME in each relevant cell type (3). Human relevance: while animal models provide strong evidence, human hearts might have differences. It is possible that pyroptosis might be even more impactful in humans.

The unique role of GSDME is to link apoptotic stimuli to pyroptotic outcomes, effectively converting what might be “silent” cell death into loud inflammatory events. This process results in a vicious cycle involving inflammation and oxidative stress, each of which feeds the other. Research in the past 5 years converges on the notion that excess or dysregulated pyroptosis is detrimental in CVD patients and that controlling it holds therapeutic promise. There is no doubt that therapeutic targeting of GSDME is a nascent yet promising field, although most of them focus on the oncology field. As of 2025, therapies specifically targeting pyroptosis in CVD patients are in preclinical development. Notably, some studies have demonstrated that active components in certain traditional Chinese medicines, such as astragaloside IV and ginsenoside Rh2, can alleviate chemotherapy-induced cardiotoxicity by inhibiting the caspase-3/GSDME pathway (123, 124). These findings not only provide a novel therapeutic approach for cardiovascular diseases but also offer a potential research direction for strategies aimed at prolonging the survival of cancer patients. Similarly, ligustrazine alleviates coronary artery calcification by inhibiting the caspase-3/GSDME-mediated pyroptosis pathway (125). These studies collectively suggest that bioactive compounds exert protective effects on the cardiovascular system and provide important theoretical support for the development of novel therapeutic strategies for cardiovascular diseases. Additionally, Xu et al. utilized an exosome delivery system to regulate the expression of GSDME, thereby successfully influencing coronary artery calcification (126). This study highlights the progress of interdisciplinary medical engineering technologies and novel diagnostic and therapeutic approaches, providing new perspectives for patient treatment. An intriguing study revealed that aerobic exercise inhibits GSDME-dependent cardiomyocyte pyroptosis, thereby effectively protecting the heart from ischemia–reperfusion injury (127).

## 6 Conclusions

In conclusion, GSDME has emerged as a crucial mediator linking regulated cell death to inflammation in the cardiovascular diseases. Through its caspase-3-dependent activation and pore-forming capacity, GSDME converts apoptotic signals into secondary pyroptosis, amplifying tissue inflammation and injury in conditions such as atherosclerosis, myocardial infarction, cardiomyopathy, and pulmonary hypertension. Both Experimental and clinical studies have suggested that GSDME has a dual role, acting as a driver of pathology and a potential therapeutic target. Continued exploration using integrated molecular, cellular, animal, and omics-based approaches will be essential to clarify its regulatory mechanisms and disease specificity. Translational efforts that modulate GSDME activation or expression may offer innovative strategies to protect cardiovascular system. If successfully applied in the clinical practice, such strategies could reduce inflammation, ultimately improving cardiovascular health, longevity, and quality of life.
